# The Effect of Salinity on Heavy Metal Tolerance in Two Energy Willow Varieties

**DOI:** 10.3390/plants14121747

**Published:** 2025-06-07

**Authors:** Kinga Drzewiecka, Zuzanna Kaźmierczak, Magdalena Woźniak, Michał Rybak

**Affiliations:** 1 Department of Chemistry, Faculty of Forestry and Wood Technology, Poznań University of Life Sciences, Wojska Polskiego 75, 60-625 Poznań, Polandmagdalena.wozniak@up.poznan.pl (M.W.); 2 Department of Water Protection, Faculty of Biology, Adam Mickiewicz University, 61-614 Poznań, Poland; m.rybak@amu.edu.pl

**Keywords:** environmental stress, metal uptake, osmotic stress, phytoremediation, *Salix* sp.

## Abstract

This study evaluated the response of two willow varieties, *Salix × smithiana* Willd. and *Salix viminalis* L. var. Gigantea, to selected heavy metals and elevated soil salinity, simulating complex environmental conditions during phytoremediation. Plants propagated from stem cuttings were cultivated in pots under field conditions in soil artificially contaminated with a mixture of Cd, Ni, Cu, Zn, and Pb salts at two concentration levels representing lower and higher guideline thresholds. Sodium chloride was added to induce salinity stress. *S. × smithiana* exhibited enhanced growth under combined metal and salinity stress, suggesting efficient tolerance mechanisms. This was reflected in elevated relative water content (RWC) and increased accumulation of Zn and Cd in shoots. In contrast, Gigantea showed growth inhibition and primarily sequestered metals in roots, indicating a stress-avoidance strategy and reduced metal translocation. While salinity alone negatively affected both varieties, its combination with metals mitigated growth reduction in *S. × smithiana*, possibly due to improved ion homeostasis or cross-tolerance. Zn and Cd displayed the highest bioconcentration and mobility. Based on bioconcentration factor (BCF) and translocation factor (TF), *S. × smithiana* appears suitable for phytoextraction, whereas *S. viminalis* var. Gigantea appears suitable for phytostabilization. These results support species-specific approaches to phytoremediation in multi-contaminant environments.

## 1. Introduction

Heavy metals, including cadmium (Cd), lead (Pb), copper (Cu), zinc (Zn), nickel (Ni), chromium (Cr), and arsenic (As), pose significant threats to both environmental and human health. They are persistent, non-biodegradable, and can accumulate in soils, adversely affecting soil-dwelling organisms, and, through agricultural uptake, they enter the food chain, thereby impacting animals and humans. Soluble forms of metals can leach from soils into surface- and groundwater, leading to bioaccumulation in aquatic ecosystems and contamination of drinking water intakes. A recent study summarized the global prevalence of heavy metal contamination estimating that approximately 14–17% of the world’s cropland (about 242 million hectares) is polluted with toxic metals, posing serious risks to food safety and human health [1]. Additionally, widespread presence of heavy metals in aquatic systems, originating from industrial and urban wastewaters, surface runoff, and deposition, is causing significant ecological and health concerns and has been recently summarized [2].

Remediation of environments polluted with heavy metals, such as the soil and water, can be achieved through physicochemical, technical, and biological methods. Biological strategies are distinguished by their minimal environmental disruption and broad public acceptance [3,4]. Among these, phytoremediation has been defined as the use of living plants to remove, degrade, or neutralize pollutants in situ from soil, surface water, or groundwater. Plants used in phytoremediation primarily include trees, shrubs, grasses, and aquatic plants, often in association with their microbiomes. To enhance the efficiency of phytoextraction and to restore balance in the remediated ecosystem, additional approaches such as soil inoculation with endophytes, plant growth-promoting rhizobacteria (PGPR), or arbuscular mycorrhizal fungi are employed. Phytoremediation utilizes the ability of plants to accumulate heavy metals from soil or water (including wastewater) in their aerial parts, which can subsequently be harvested and removed. It also exploits the phenomenon of metal stabilization within the rhizosphere [4].

Climate change has intensified the frequency and severity of droughts globally, posing significant risks to water resources, ecosystem stability, and agriculture [5]. Changes in precipitation patterns and increased evapotranspiration have led to severe soil moisture deficits, negatively affecting crop productivity. Particularly, Mediterranean areas, southwestern North America, southern Africa, and parts of South Asia are suffering prolonged drought events [6,7]. Also in other regions, drought has become more prevalent and exacerbated by rising temperatures. Drought may also pose a significant challenge to soil phytoremediation projects, as a water deficit can impair plant growth, reduce biomass production, and limit the uptake and translocation of pollutants, thereby decreasing the efficiency of the remediation process [8]. Drought events are closely linked to increasing soil salinization, particularly in arid and semi-arid regions [9,10]. Reduced precipitation and increased evapotranspiration during droughts lead to a decline in soil moisture and groundwater recharge, which elevates salt concentration in the root zone. Moreover, in irrigated agricultural areas, prolonged droughts often prompt the use of saline or marginal-quality water for irrigation, exacerbating salt accumulation. This process not only reduces crop productivity but also deteriorates soil structure and fertility and contributes to land degradation [11].

Recent studies have highlighted the potential of (bio)energy plants, particularly species from the Salicaceae family, namely willows (*Salix* spp.) and poplars (*Populus* spp.), in biological remediation programs [12,13,14]. Energy trees and shrubs can be cultivated in short-rotation coppice (SRC) systems, exhibit significant resistance to pests and diseases, accompanied with high tolerance to heavy metals, and contribute positively to the environment by enhancing soil organic matter content and improving soil structure [12]. Furthermore, their extensive root systems facilitate the effective uptake and immobilization of heavy metals, making them suitable candidates for phytoremediation purposes [12,15]. This was confirmed in a previous large-scale study on the efficiency of Cu, Pb, and Zn phytoextraction by 145 willow taxa during cultivation in area affected by industrial activity [16]. However, climate change highlights the urgent need for adaptive strategies in water management as well as in agricultural planning and phytoremediation projects. Thus, the aim of this study was to investigate the effect of increased soil salinity on heavy metal toxicity to two varieties of energy willow, particularly on plant growth (biomass yield) and metal uptake abilities. Understanding the interactions between soil salinity and heavy metal toxicity is essential for optimizing the application of energy willow in metal-polluted and marginal lands under future climate scenarios.

## 2. Results

### 2.1. Biomass Parameters

**Figure 1:**
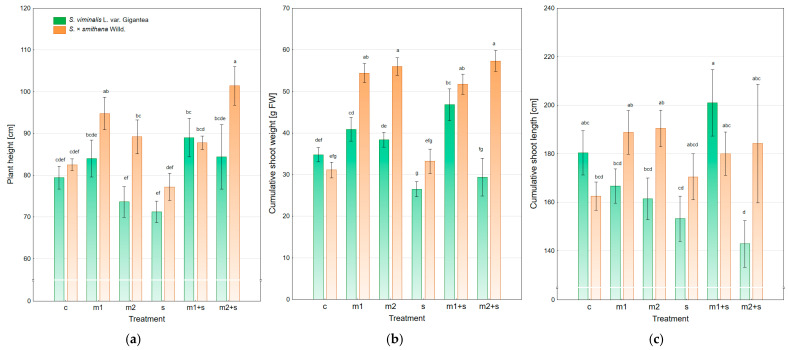
Biomass parameters of energy willow under different experimental treatments, (**a**) plant height; (**b**) cumulative shoot weight; (**c**) cumulative shoot length (c—control; m—application of a heavy metal mixture at lower (1) and higher (2) concentrations; s—salinity); mean ± standard error (*n* = 5). Different letters indicate significant differences between means according to Tukey’s HSD test at α = 95% for “variety × treatment” fixed effect.

The mean height of control plants for both analyzed willow varieties was comparable, amounting to 79.4 cm and 82.5 cm for *Salix viminalis* var. Gigantea and *Salix × smithiana*, respectively (Figure 1a). Significant differences in plant height between the two varieties were observed across experimental treatments. *S. × smithiana* generally achieved greater height than *S. viminalis* var. Gigantea in nearly all treatment variants, with significant differences noted under the m2 and m2+s treatments (by 21.2% and 20.1%, respectively). The lowest plant height in both varieties was recorded under salinity stress (NaCl addition), particularly for the Gigantea variety (71.2 cm). The application of the metal mixture significantly differentiated the two willow varieties. In the case of *S. × smithiana*, shoot growth was stimulated relative to the control by 14.9% and 8.1% under m1 and m2 treatments, respectively. In contrast, in *S. viminalis* var. Gigantea, plant height was similar to the control in m1, and lower in m2. The combined application of heavy metals and NaCl (m + s) alleviated the negative effect of salinity and stimulated shoot elongation, even in comparison to the control. This effect was particularly significant for *S. × smithiana*, which showed the highest average height in the m2+s variant (101.4 cm), representing increases of 22.9%, 13.7%, and 31.5% relative to the control, m2, and salinity treatments, respectively.

The cumulative fresh shoot biomass of energy willow showed significant differences between the tested varieties and experimental treatments (Figure 1b). *S. × smithiana* showed higher biomass values (mean 47.3 g) compared to *S. viminalis* var. Gigantea (36.1 g), which is consistent with previous observations regarding plant height. The highest shoot biomass (57.3 g) was recorded for *S. × smithiana* under m2+s treatment, representing increases of 22.9%, 13.7%, and 31.5% compared to the control, m2, and salinity treatments, respectively. Other treatment combinations (m1, m2, and m1+s) also had a stimulatory effect on this variety. In contrast, Gigantea exhibited weaker responses, i.e., biomass produced under m2 and m2+s treatments was lower than in the control, although a slight positive effect was noted under m1+s (46.8 g). Salinity alone (s) markedly reduced shoot biomass in both varieties, especially in *S. viminalis* var. Gigantea (26.5 g). The observed trends aligned with plant height results, i.e., *S. × smithiana* consistently achieved higher values for both parameters under similar conditions.

The cumulative shoot length of energy willow also varied between the tested varieties and environmental treatments. *Salix × smithiana* achieved a slightly higher average value (179.4 cm) than *S. viminalis* var. Gigantea (167.6 cm), consistent with earlier trends observed for plant height and biomass. The highest total shoot length (201.0 cm) was recorded for the Gigantea variety under the m1+s treatment, representing an exception compared to other growth parameters, where *S. × smithiana* generally dominated. However, for the same variety, the lowest result (142.8 cm) was observed in the m2+s variant, indicating a negative impact of the higher metal dose combined with salinity. In contrast, *S. × smithiana* exhibited comparable shoot length across the m1, m2, and m2+s treatments (188.8–190.4 cm), suggesting greater consistency in response to stress factors. While salinity alone exerted a growth-inhibiting effect on both varieties, it was less pronounced than for previous parameters. Compared to plant height and biomass, the differences in cumulative shoot length were less pronounced, and the advantage of *S. × smithiana* was weaker. This may suggest that shoot length is a more variable or less sensitive parameter in response to the tested environmental factors.

### 2.2. Relative Water Content in Leaves

**Figure 2:**
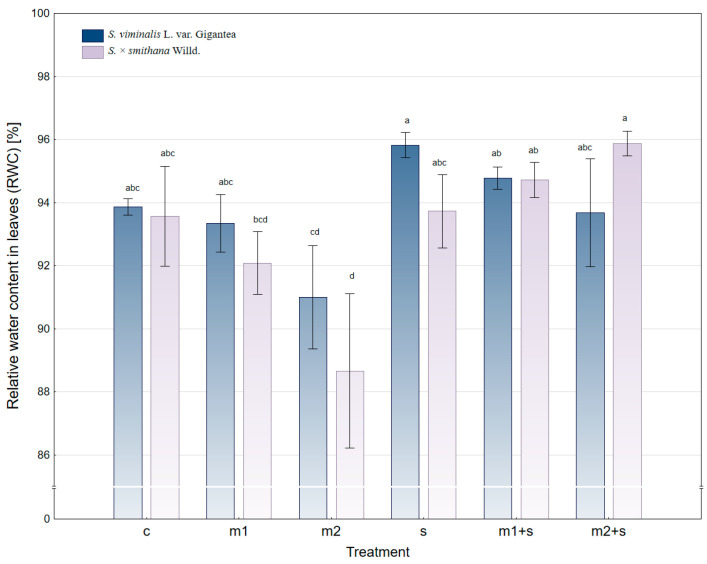
Relative water content (RWC) in leaves of energy willow under different experimental treatments (c—control; m—application of a heavy metal mixture at lower (1) and higher (2) concentrations; s—salinity); mean ± standard error (*n* = 5). Different letters indicate significant differences between means according to Tukey’s HSD test at α = 95% for “variety × treatment” fixed effect.

The RWC in the leaves of energy willow differed significantly between the studied varieties and pollution variants (Figure 2). The average RWC across the experimental population was 93.42%, with *S. viminalis* var. Gigantea exhibiting slightly higher values (93.74%) compared to *S. × smithiana* (93.10%). Control and low-metal treatments (m1) were characterized by intermediate RWC levels, while m2 treatments reduced RWC in both cultivars and more markedly in *S. × smithiana*. Among the environmental treatments, the highest RWC was observed for the combination of heavy metals with salinity (m2+s), particularly in *S. × smithiana*, where the value reached 95.88%. Conversely, the lowest RWC was recorded in the same cultivar under the m2 treatment (88.66%), indicating the negative impact of elevated heavy metal levels in the absence of salinity, likely imposing stress on the plant’s water management.

In *S. viminalis* var. Gigantea, the RWC values were more stable across treatments. The highest RWC for this variety was also found in the salt-amended variants (s, m1+s, and m2+s), confirming the mitigating effect of salinity on metal-induced stress observed in both cultivars.

### 2.3. Metal Content in Willow Organs

#### 2.3.1. Leaves

**Figure 3:**
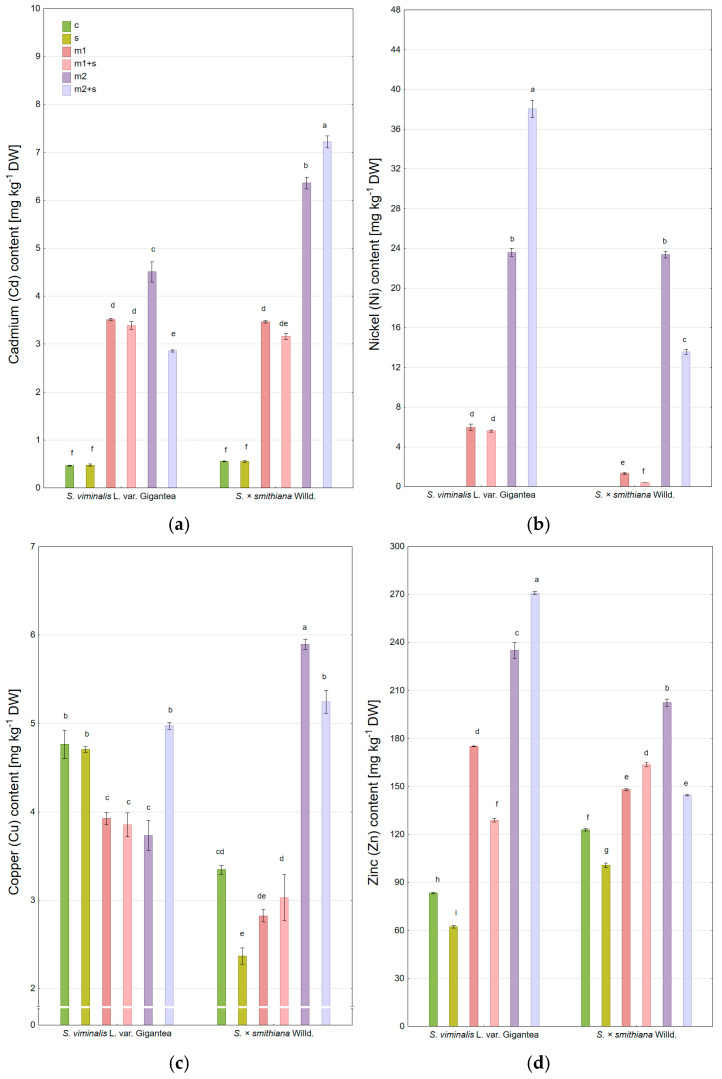
Heavy metal content in willow leaves under experimental treatments, (**a**) cadmium (Cd); (**b**) nickel (Ni); (**c**) copper (Cu); (**d**) zinc (Zn) (c—control; m—addition of metal mixture at lower (1) and higher (2) levels; s—salinity). DW—dry weight; mean ± standard error (*n* = 3). Different letters indicate significant differences between means according to Tukey’s HSD test at α = 95% for “variety × treatment” fixed effect.

The content of cadmium (Cd) in the leaves of control plants and those subjected to salinity remained low and comparable (~0.5 mg kg^−1^) (Figure 3a). A significant increase in Cd content was observed in both willow varieties at the lower metal dose (m1), with average levels around 3.4 mg kg^−1^, regardless of the presence of additional salinity. At the higher dose (m2), Cd was intensively accumulated in the leaves of *S. × smithiana*, and salinity further enhanced this accumulation (6.4 and 7.2 mg kg^−1^, respectively). In contrast, for the Gigantea cultivar under m2 treatment, Cd level was significantly lower (4.5 mg kg^−1^) and decreased further in the combined metal–salinity treatment (m2+s) to 2.9 mg kg^−1^.

Nickel (Ni) was not detected in the control or salinity-only treatments (Figure 3b). Under the m1 treatment, Gigantea showed a higher Ni accumulation than *S. × smithiana*, and the Ni content (~5.6 mg kg^−1^) was unaffected by salinity. At the higher dose (m2), Ni accumulation increased substantially in both cultivars (~23 mg kg^−1^), while the addition of salinity either increased (Gigantea) or decreased (*S. × smithiana*) Ni uptake by approximately 50% relative to the m2 variant.

For Gigantea, copper (Cu) content in leaves under modified soil conditions did not exceed that observed in control plants (4.8 mg kg^−1^ for Gigantea), and Cu concentrations were lower than the control in treatments m1, m1+s, and m2 (Figure 3c). In *S. × smithiana*, only the m2 treatment led to increased Cu accumulation in leaves (up to 5.9 mg kg^−1^) compared to the control (3.5 mg kg^−1^), though this was slightly reduced (~10%) when salinity was also applied.

Zinc (Zn) concentrations in control leaves were 83.4 and 123 mg kg^−1^ for Gigantea and *S. × smithiana*, respectively, with salinity-only treatment (s) reducing Zn uptake in both cultivars (Figure 3d). Gigantea accumulated more Zn than *S. × smithiana* at both metal concentrations, and the effect of salinity on Zn uptake varied depending on the metal dose and cultivar—being either inhibitory or stimulatory. The highest Zn content was observed in Gigantea under the m2+z treatment (271 mg kg^−1^).

Lead (Pb) was not detected in the willow leaves in any of the experimental treatments.

#### 2.3.2. Stems

**Figure 4:**
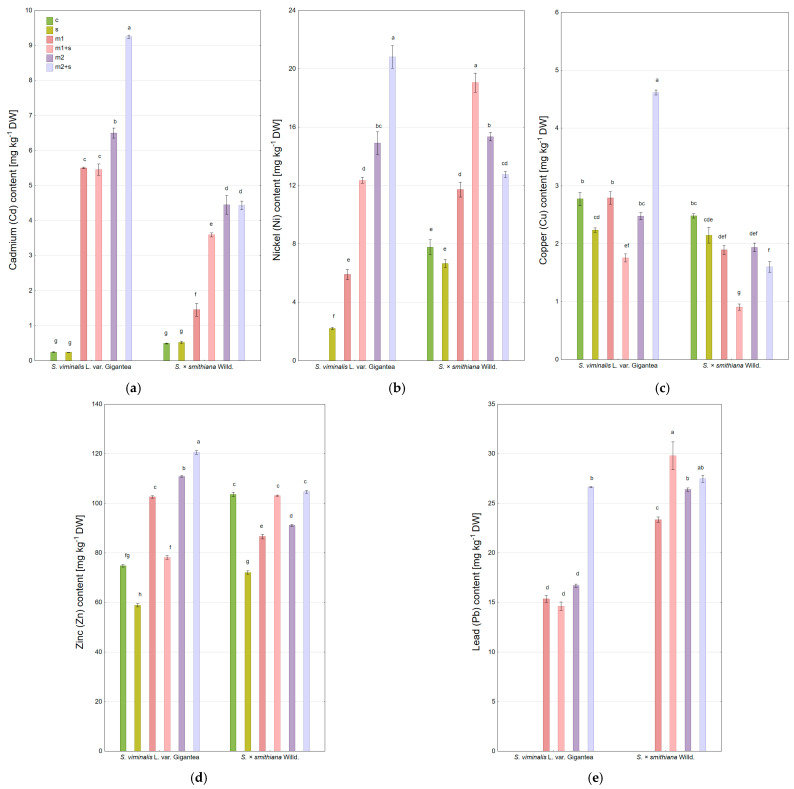
Heavy metal content in willow stems under experimental treatments, (**a**) cadmium (Cd); (**b**) nickel (Ni); (**c**) copper (Cu); (**d**) zinc (Zn); (**e**) lead (Pb) (c—control; m—addition of metal mixture at lower (1) and higher (2) levels; s—salinity). DW—dry weight; mean ± standard error (*n* = 3). Different letters indicate significant differences between means according to Tukey’s HSD test at α = 95% for “variety × treatment” fixed effect.

The average Cd content in willow stems was comparable to the leaves (3.5 and 3.0 mg kg^−1^ DW, respectively). Control and salinity-only treatments exhibited very low Cd levels (below 0.5 mg kg^−1^ DW) (Figure 4a). Following metal additions to the soil, Gigantea accumulated Cd in stems to higher content than *S. × smithiana*, particularly at a higher metal dose (m2). The additional salinity enhanced Cd accumulation depending on both the metal dose and the willow variety (in the m2 variant for Gigantea and m1 for *S. × smithiana*). The highest Cd concentration was recorded in stems of Gigantea in m2+s variant (9.3 mg kg^−1^ DW), while for *S. × smithiana* the accumulation was weaker and reached 4.4 mg kg^−1^ DW.

Similar patterns were observed for nickel (Ni), with the highest concentration also recorded in the m2+s variant (20.8 mg kg^−1^ DW for Gigantea), while Ni was undetectable in stems of control plants (Figure 4b). Across all treatments, the average Ni content in stems was comparable to that in leaves (10.8 vs. 9.3 mg kg^−1^ DW). Under the lower metal dose (m1), higher Ni accumulation was observed in *S. × smithiana*, whereas under the higher dose (m2), both varieties showed significantly higher and comparable Ni contents. In Gigantea, salinity further enhanced Ni uptake to stems, whereas in *S. × smithiana*, this effect was only observed at the lower Ni dose (m1); at the higher dose (m2), salinity actually reduced Ni content.

The content of Cu in stems were variable but generally lower than those of the other metals and lower than Cu levels in leaves (2.3 vs. 4.1 mg kg^−1^ DW, respectively) (Figure 4c). Addition of Cu to soil did not cause its elevated accumulation in stems, and in *S. × smithiana*, Cu content was even lower than in control plants. In all cases, salinity reduced Cu content in stems, with the exception of the m2+s treatment in Gigantea, which exhibited the highest Cu content (4.61 mg kg^−1^ DW).

Accumulation of Zn in stems was significantly lower than in leaves (92.2 and 153.2 mg·kg^−1^ DW, respectively). In both varieties, salinity markedly decreased Zn content compared to controls (Figure 4d). Metal addition to the soil only promoted Zn accumulation in Gigantea, reaching 120.6 mg kg^−1^ DW in the m2+s treatment. At the lower metal dose (m1), salinity had a negative impact on Zn uptake. In *S. × smithiana*, Zn contents in metal-amended treatments were lower than in stems of control plants, and additional salinity slightly increased Zn uptake, though only to levels comparable with the control.

The level of Pb was undetectable in stems of control and salinity-only treatments (Figure 4d). Metal addition led to Pb accumulation in stems, with higher contents observed in *S. × smithiana*. In Gigantea, Pb levels were lower and similar under both metal doses, with a significant increase only under the m2+s treatment. In *S. × smithiana*, salinity also elevated Pb content, with a stronger effect observed at the lower metal dose (m1). Consequently, the highest Pb content (29.8 mg kg^−1^ DW) was recorded in *S. × smithiana* under the m1+s treatment.

#### 2.3.3. Roots

**Figure 5:**
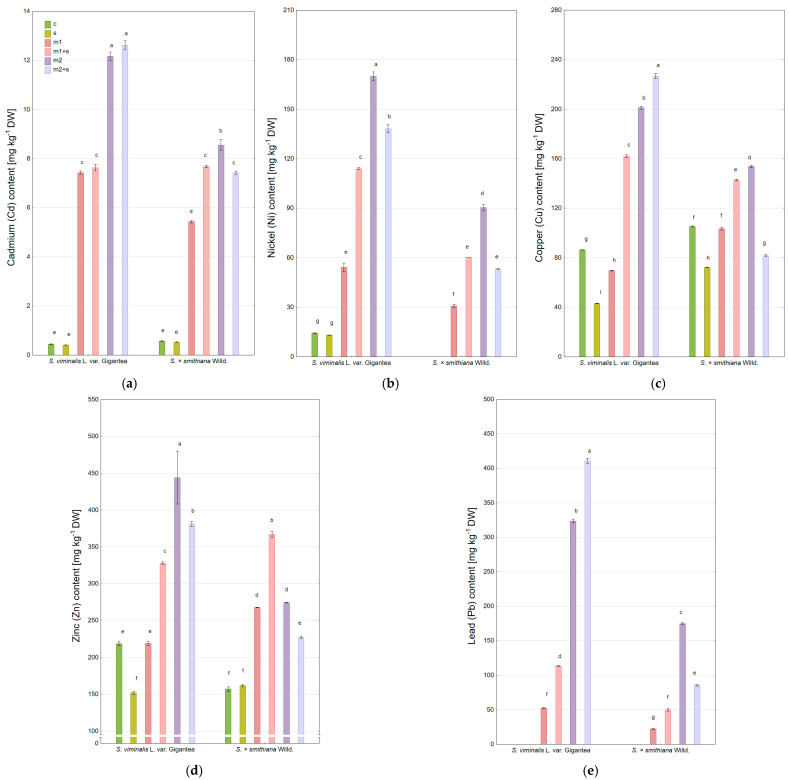
Heavy metal content in willow roots under experimental treatments, (**a**) cadmium (Cd); (**b**) nickel (Ni); (**c**) copper (Cu); (**d**) zinc (Zn); (**e**) lead (Pb) (c—control; m—addition of metal mixture at lower (1) and higher (2) levels; s—salinity). DW—dry weight; mean ± standard error (*n* = 3). Different letters indicate significant differences between means according to Tukey’s HSD test at α = 95% for “variety × treatment” fixed effect.

The average concentrations of the analyzed metals in willow roots were higher than in aboveground organs, particularly for Cu, where levels were approximately 60 times greater, while the smallest difference was observed for Cd, with root concentrations less than twice those in stems (Figure 5a–d).

In contrast to other organs, a clearer pattern of metal uptake can be noted. Increasing the metal dose generally resulted in higher metal accumulation in roots, with the exception of Zn in *S. × smithiana*, where similar levels were determined at both applied doses (Figure 5d). *S. × smithiana* exhibited lower average root concentrations compared to Gigantea, except for Cu and Zn at m1 treatment. Moreover, in *S. × smithiana* and at the higher metal dose, salinity consistently reduced metal uptake from the substrate, while at the lower dose, it enhanced uptake. In Gigantea, a similar salinity effect was observed for Zn and Ni, whereas for Cu and Pb, salinity increased metal accumulation in roots at both metal doses. Only in the case of Cd did salinity have no significant effect on metal accumulation in roots, regardless of the applied Cd dose (Figure 5a).

Similar as in stems and leaves, Pb was not detected in the roots of control plants or those treated with salinity alone (Figure 5e).

#### 2.3.4. Metal Accumulation Ratios

**Figure 6:**
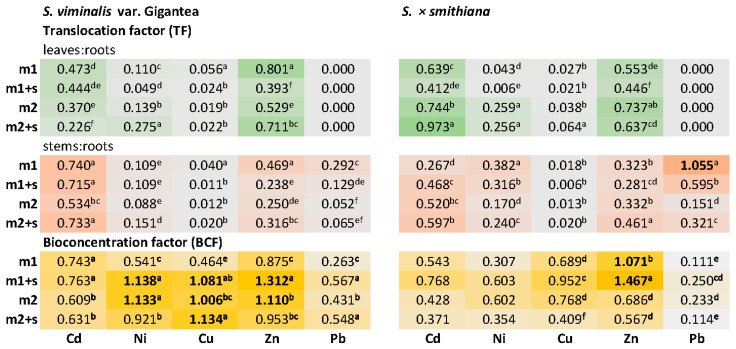
**A heatmap presenting** translocation factor (TF) and bioconcentration factor (BCF) values under experimental treatments (m—addition of metal mixture at lower (1) and higher (2) levels; s—salinity). Different letters indicate significant differences between means according to Tukey’s HSD test at α = 95% for “variety × treatment” fixed effect; values ≥1 indicated in bold.

The highest translocation factor (TF) values were calculated for Zn and Cd; however, they did not exceed 1 (Figure 6). In the case of Pb, its translocation was restricted to stems only, and in *S. × smithiana* the TF values reached 1.071 at the lower metal dose (m1) with a reduction by half when salinity was simultaneously applied. The highest bioconcentration factor (BCF) values were noted for Zn, and the lowest values were noted for Pb (Figure 6). *S. × smithiana* strongly accumulated Zn in roots at m1, and salinity elevated the BCF value. For the Gigantea variety, the BCF values exceeding 1 were observed for Ni, Cu, and Zn, with salinity inducing metal uptake to roots at m1, and at m2 the induction effect was noted only in the case of Cu.

### 2.4. Principle Component Analysis (PCA)

**Figure 7:**
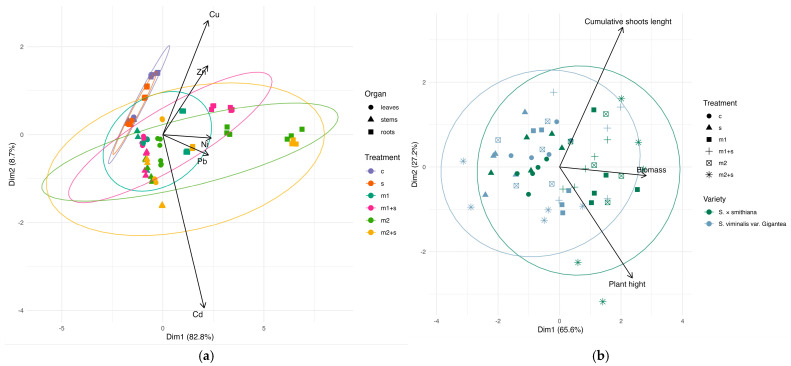
Principal component analysis (PCA) of metal accumulation (**a**) and biomass traits (**b**) of investigated willow varieties in experimental variants (c—control; m—addition of metal mixture at lower (1) and higher (2) levels; s—salinity).

The first PCA was conducted to assess patterns of heavy metal accumulation in different plant organs under experimental treatments (Figure 7a). The first two principal components (dim1 and dim2) jointly explained 91.5% of the total variance, 82.8% and 8.7%, respectively. The dim1 showed strong positive correlations with all analyzed elements, indicating that it represents the primary axis of variation associated with overall heavy metal accumulation in plant tissues. In contrast, Cd exhibited a strong negative correlation with dim2, suggesting that this second component captures specific variation related to Cd accumulation relative to the other elements. The PCA results reflect the influence of both treatments and variation in accumulation across organs. The control and salinity treatments clustered near the origin of the coordinate system, indicating lower levels of metal accumulation. They were relatively similar to one another, suggesting a similar response without metal addition. In contrast, treatments involving metals were shifted toward higher dim1 values, with the strongest response observed for combined salinity and metal treatments, indicating elevated levels of heavy metal accumulation. Notably, this accumulation was primarily observed in the roots. Both the leaves and, especially, the stems were more closely associated with Cd accumulation, as indicated by the direction of the Cd vector.

The second PCA analysis was performed to evaluate the variability of growth-related traits in willow varieties (Figure 7b). The first two principal components jointly accounted for 92.8% of the total variance, with contributions of 65.6% and 27.2%, respectively. The dim1 was influenced by all three measured traits, with biomass showing the strongest contribution, followed by plant height and cumulative shoot length. This indicates that dim1 reflects a general gradient of plant productivity. The dim2 was primarily associated positively with cumulative shoot length and negatively with plant height, suggesting that it represents differences in growth strategy between shoot elongation and the vertical growth of the main stem. The PCA results revealed moderate differentiation among the analyzed willow varieties. Samples of *S. × smithiana* were mainly clustered on the positive side of dim1, suggesting slightly higher overall productivity for this variety. Treatment effects were also evident, particularly in the metal-amended variants, which shifted sample positions toward higher dim1 values, indicating enhanced biomass production. In contrast, samples from the control and salinity treatments clustered closer to the origin of the coordinate system, revealing a limited growth response under these conditions.

## 3. Discussion

### 3.1. Growth Response

Metals such as Cd, Ni, Cu, Pb, and Zn can cause negative effects in plants when exceeding phytotoxicity threshold, including disruption of nutrient uptake, enzyme activity, photosynthesis, and induction of oxidative stress [17,18]. However, certain plant species, particularly hyperaccumulators and fast-growing woody perennials like willow and poplar, are capable of maintaining or even enhancing biomass production in metal-polluted soil [14,19]. In the present study, *S. × smithiana* exhibited growth stimulation following metal treatments at given levels corresponding to guideline values of soil pollution. The observation was consistent with the hormesis effect, an adaptive response in which low or moderate levels of stressors enhance plant growth [20]. This could involve the activation of specific cellular pathways, such as the upregulation of protective genes or the modification of plant metabolism. As a consequence, hormesis triggers beneficial physiological and biochemical changes in plants, such as enhanced antioxidant production, increased defense mechanisms, or improved nutrient uptake [20].

The increased plant height and fresh shoot biomass in *S. × smithiana* under m2+s treatment was particularly notable. This indicates that salinity, at the applied concentration, may facilitate the ability of willow to tolerate heavy metal stress, potentially by influencing ion balance or enhancing root membrane stability [21,22]. Enhanced shoot elongation and biomass were previously observed in willow species exposed to combined salt and heavy metal stress [23]. Conversely, *S. viminalis* var. Gigantea was sensitive to the applied conditions, especially under m2 and m2+s treatments, where a decline in growth was observed. Changes in growth rate as a response to metal may result directly from its toxicity but also from an adaptive reaction and energy allocation. Plants under abiotic stress may redirect metabolic resources toward stress mitigation rather than biomass production.

The enhanced growth of *S. × smithiana* under metal and combined metal–salinity treatments confirms previous findings on willow capacity to sustain biomass production under abiotic stress [24]. *S. × fragilis* not only tolerated Zn and Cu exposure under drought conditions but also exhibited sex-dependent differences in growth, with female plants maintaining growth and biomass yield.

### 3.2. Water Content as Physiological Response

Relative water content (RWC) is a key indicator of plant water status and stress tolerance [25]. In the present study, RWC was most affected in plants growing at a higher pollution level, particularly in *S. × smithiana*, while the highest RWC value was noted in this variety under m2+s co-treatment. This indicates that salinity, in combination with heavy metals, may induce physiological responses such as osmolyte accumulation to retain water and maintain cell turgor pressure, as well as osmoprotectants of antioxidant activity such as proline to mitigate oxidative stress [26]. An increased proline accumulation was previously found in *S.× fragilis* in response to Zn and Cu under drought conditions in hydroponic experiment [24].

In contrast to *S. × smithiana*, Gigantea maintained relatively stable RWC values across treatments. This reflects efficient root water uptake mechanisms even under given stress conditions. However, this was not reflected in biomass yield under salinity and metal co-treatment, which may suggest that the response was limited to water regulation, rather than metal detoxification or antioxidant activity.

### 3.3. Mechanism of Plant Reaction Behind Salinity Effect on Heavy Metal Toxicity

The enhanced tolerance of *S. × smithiana* to heavy metals and salinity stress can be attributed to a combination of physiological, biochemical, and molecular mechanisms that enable the plant to maintain growth under adverse soil conditions. One of the primary strategies is the upregulation of antioxidant defense systems, including enzymatic antioxidants such as superoxide dismutase (SOD), peroxidase (POD), and catalase (CAT), which protect cellular structures from oxidative damage [8]. These enzymes play a crucial role in detoxifying reactive oxygen species (ROS) generated under both metal and osmotic stress conditions. In response to heavy metals, plants produce metal-binding ligands such as phytochelatins and metallothioneins, which chelate metal ions and facilitate their sequestration in vacuoles, thus reducing their cytotoxic effects [27]. Another important mechanism is the regulation of ion transport and homeostasis. Plants activate specific ion transporters, which modulate the uptake, compartmentalization, and long-distance transport of metal ions [18]. In parallel, exposure to salinity induces the accumulation of osmoprotectants, including proline, soluble sugars, and polyols, which stabilize proteins and membranes and help maintain cellular osmotic balance [28]. The interaction between heavy metal and salinity stress likely involves crosstalk between signaling pathways, including those mediated by abscisic acid (ABA), calcium ions, and MAP kinases. These shared pathways allow plants to integrate multiple stress signals and lead to appropriate gene expression as a joint response [29]. Such cross-tolerance mechanisms may explain the improved growth and biomass accumulation observed in *S. × smithiana* under combined stress conditions, highlighting its capacity to withstand complex environmental challenges. These adaptations suggest that *S. × smithiana* possesses efficient mechanisms that confer resilience to heavy-metal-polluted and saline soils, proving its utility for phytoremediation in degraded environments.

### 3.4. Metal Uptake and Accumulation Patterns

The accumulation of heavy metals in plant organs differed significantly between the two willow genotypes and among the analyzed organs. Previous studies also documented significant differences in heavy metal accumulation and translocation patterns among *Salix* genotypes resulting from their genetic variability [30,31,32]. Willow roots accumulated the highest contents of metals, particularly Cu and Pb, which aligns with previous studies indicating that roots serve as a primary barrier against systemic metal toxicity [33], and metal sequestration within roots is a common strategy in many plant species to reduce damage to photosynthetic tissues [34,35]. However, it also limits the plant’s potential for phytoextraction of metals from polluted soil. Particularly, *S. viminalis* var. Gigantea exhibited higher root accumulation of most metals, especially under m2+s, indicating its potential for metal phytostabilization, even at increased salinity. Metal uptake patterns observed for Gigantea confirm previous findings for *S. × fragilis*, which accumulated Cu primarily in the roots, thus limiting shoot translocation and mitigating oxidative stress [24]. In other studies, the accumulation of metals (Ni, Pb, Cu, and Cd) varied between genotypes of *S. alba* and *S. viminalis*; however, in general the content of Ni and Pb was the highest in the roots, while Cu was accumulated by all plant organs, and Cd was translocated to the leaves. In consequence, differences in metal tolerance and accumulation ratios between willow genotypes are a key factor for plant selection in soil phytoremediation strategies including phytostabilization or phytoextraction [35].

In contrast, *S. × smithiana* showed higher Cd accumulation in leaves, mainly under m2 and m2+s treatments. Although Cd is generally mobile within the plant, its accumulation in leaves can lead to negative effects due to interference with photosynthesis and enzyme function [36]. The higher values of the translocation factor (TF) for Cd and Zn, particularly in *S. × smithiana*, suggest a greater capacity for and tolerance to metal accumulation in the aboveground organs, which is a desirable trait in phytoextraction [37,38]. As previously documented, particular willow clones are capable of high foliar accumulation of Cd and Zn with only mild phytotoxicity symptoms attributable to excess Zn [37]. This was consistent with other studies which demonstrated that numerous *Salix* clones could easily translocate Cd and Zn from soil to aboveground organs, while the rate of Pb translocation was limited [30]. Similarly, in our study, considering BCF values, Pb was accumulated to the least extent, and its translocation to leaves was not detected. Other studies on willows, including *S. × smithiana*, also reported limited translocation of Pb to aboveground organs, with higher amounts retained in the roots [39]. A meta-analysis of 194 publications for the period 1975–2016 on heavy metal accumulation capacities of different willow genotypes concluded that willows can be considered as accumulators of Cd (in twigs and leaves), Pb (in roots and twigs), and Zn (in twigs), while lower soil pH promotes Cd accumulation in stems [32]. Furthermore, in an inter-species comparison of studies on willow, poplar, and maize metal accumulation properties, *S. × smithiana* accumulated the highest contents of Cd and Zn in biomass mainly in the leaves [40].

The accumulation patterns may also be influenced by the interaction of metal ions with salinity under combined stress conditions. Particularly, competition between Na^+^ and heavy metal cations could lead to their reduced or enhanced uptake. As documented, the salinity may influence the uptake of heavy metals (Zn, Cu, and Cd), with the effects varying depending on the plant species and salinity levels [28]. Specifically, moderate salinity levels enhanced metal accumulation in some species, while higher salinity levels activated ionic exclusion mechanisms that reduced the uptake of both Na^+^ and heavy metals [41]. Salts modify heavy metal mobility in soil and the effect depends significantly on the salt type, its concentration, and the heavy metal level. Among several salts applied in a comparative study, sodium chloride significantly increased the mobilization of Cd > Pb > Cu > Zn [42]. These complex interactions underline the importance of investigations on stress combinations rather than single-factor experiments.

### 3.5. Implications for Phytoremediation of Metal-Polluted Soil

Research on different willow genotypes revealed differences in the bioconcentration factor (BCF) and translocation factor (TF) values depending on the metal, its concentration, and the cultivation conditions. For example, *S. viminalis* clones showed high Cu and Zn accumulation in aerial parts (Cu BCF up to 2.2; Zn BCF ~3.5), whereas *S. purpurea* varieties demonstrated lower TF values, indicating more root-based sequestration [31]. In another study [35], *S. alba* clones had a higher potential for the phytostabilization of Cu and Cd, as well as the phytoextraction of Cd, compared with *S. viminalis*. These findings highlight the importance of genotype selection in phytoremediation strategies.

In the present study, the values of BCF and TF suggest that *S. × smithiana* is more suited for phytoextraction, especially for Zn and Cd, due to higher shoot accumulation rates and higher biomass production. The high accumulation capacity of *S. × smithiana* clones towards Zn was previously documented in a hydroponic screening study of twenty willow genotypes [43]. Conversely, *S. viminalis* var. Gigantea, which accumulated metals primarily in the roots, may be more suitable for phytostabilization approaches. Moreover, the ability of *S. × smithiana* to maintain high growth rates and water content under stress conditions indicates efficient tolerance mechanisms. Such species are particularly valuable in phytoremediation projects at multi-contaminant sites, such as former industrial areas, mine tailings, or agricultural land affected by metal pollution.

## 4. Materials and Methods

### 4.1. Plant Material and Experiment Setup

The one-year-old dormant shoot cuttings (stem segments, 20 cm long) of two energy willow varieties (*Salix viminalis* L. var. Gigantea and *Salix × smithiana* Willd) were purchased in late February 2024 from willow plantations located in southern Poland and were stored under refrigerated conditions (at 4 °C and 70% relative humidity) until the start of the experiment in early April 2024. Uniform, healthy cuttings of both willow varieties, standardized by stem diameter, were placed in square production pots (11 × 21.5 cm) filled with 1 kg of a 3:1 (*w*/*w*) mixture of garden soil and deacidified peat, at a moisture content of 70 ± 2.7%. One cutting was planted per pot, positioned centrally with approximately 5 cm (2–3 buds) above the substrate surface. The plants were cultivated under natural conditions and monitored using a Tempest Weather Station located in the city of Poznań (52°35′82″ N, 17°02′12″ E) (Table 1). On rainless days, the plants were watered in the evening using rainwater collected from a retention reservoir situated adjacent to the experimental site.

After six weeks of cultivation, when new shoots reached a height of approximately 20 cm, a selection was carried out to obtain uniform plant material. Subsequently, a mixture of heavy metals was introduced into the soil using solutions of their soluble salts, applied at two concentration levels established according to the guidelines of the Finnish Ministry of the Environment, as previously adopted [44] (Table 2). The Finnish legislation defines standard values that approximate regulatory systems in Europe and India for the assessment of soil pollution and the need for remediation. Higher guideline values are designated for industrial and transportation areas, whereas lower values are intended for other land-use types. Exceeding these thresholds indicates a pollution level that poses an ecological or health risk, thereby necessitating appropriate remediation measures.

The heavy metals, i.e., cadmium (Cd), lead (Pb), nickel (Ni), zinc (Zn), and copper (Cu), were applied in the form of nitrate salts: Cd(NO_3_)_2_ × 4H_2_O, Pb(NO_3_)_2_, Ni(NO_3_)_2_ × 6H_2_O, Zn(NO_3_)_2_ × 6H_2_O, and Cu(NO_3_)_2_ × 3H_2_O. The salts were dissolved in distilled water to prepare a mixture of metal ions. Based on the percentage of soil dry matter and the weight of moist soil per pot, solutions were prepared to achieve either the lower (m1) or higher (m2) guideline values for each metal (Table 2). The solutions were applied to the soil surface in a volume of 100 mL per pot to ensure uniform distribution of metals throughout the soil profile.

To induce osmotic stress (reduce water availability independently of atmospheric precipitation), a sodium chloride (NaCl; BioXtra, ≥99.5%) solution was added to the soil at a rate of 2.23 g per kg of soil dry matter (s), corresponding to an electrical conductivity of 1000 μS/cm above the baseline salinity of the substrate. The experimental design included six treatment variants for each of the two energy willow varieties, i.e., control, m1, m2, s, m1+s, and m2+s.

The pots with plants were placed in perforated polypropylene (PP) crates lined with polyethylene (PE) foil (one experimental variant per crate; five plants per variant) to prevent leaching of metals and salt from the pots to protect the ground from contamination. The crates were set on a wooden pallet, which was rotated twice a week to compensate for the influence of light direction on plant growth. After six weeks of cultivation (at the end of June), the plants were harvested and subjected to biomass parameter analysis. During the experiment, each willow cutting developed 2 to 4 new, unbranched shoots, with the majority (78.3%) producing 2 shoots. The term “cumulative shoot length” was evaluated as the sum of the lengths of all newly developed shoots on a single plant, while “cumulative shoot weight” denotes the total fresh biomass of these shoots.

Samples of individual organs (leaves, stems, and roots) were collected for laboratory analyses. The plants were removed from the pots with the soil mass densely penetrated by roots. The root systems were collected by gently shaking off the adhering bulk soil. Subsequently, the roots were thoroughly rinsed multiple times with tap water to eliminate residual soil particles, followed by a final wash with distilled water to ensure the removal of remaining contaminants.

### 4.2. Relative Water Content of Leaves

To determine the relative water content (RWC) in leaves, freshly collected leaves were weighed immediately after sampling (fresh weight = FW) and then placed in sealed zip-lock bags with distilled water, with their petioles submerged. After 24 h of incubation in darkness, the leaves were reweighed (turgid weight = TW) and subsequently dried at 105 °C to a constant weight (dry weight = DW). The RWC value was calculated using the following equation [45]:

$$RWC\;\left(\%\right)=\left[\frac{\left(FW-DW\right)}{\left(TW-DW\right)}\right]\times 100$$

### 4.3. Metal Content in Willow Organs and Uptake Ratios

The concentrations of metals (Cd, Cu, Ni, Pb, and Zn) in willow leaves, stem, and roots were determined using atomic absorption spectrometry (AAS). Plant material was dried at 70 °C to a constant weight and then ground using a laboratory mill. From the homogenized sample, 0.5 g (±0.1 mg) was weighed and subjected to acid digestion in Teflon tubes using nitric acid (HNO_3_) (Sigma Aldrich, Steinheim, Germany) in a microwave digestion system (Mars Xpress, CEM International Corporation, Matthews, NC, USA) for 30 min at 2450 MHz. The resulting solution was quantitatively transferred to volumetric flasks and brought to a final volume of 50 mL with demineralized. Metal concentrations were measured using an AA280 FS/Z AA atomic absorption spectrometer (Agilent Technologies, Santa Clara, CA, USA) with flame atomization. For each element, calibration curves were prepared based on freshly prepared standard solutions made from certified stock solutions of 1000 mg/L (Sigma Aldrich, Steinheim, Germany), with five replicates per curve. The results were expressed in mg/kg of dry matter (DM) as mean values from three measurements.

Based on the obtained results of metal content, the translocation factor (TF) was calculated as a ratio of metal content in the leaves or the stem to the roots. Additionally, the bioconcentration factor (BCF) was calculated as a ratio of metal content in the roots to its initial concentration in the soil [46,47].

### 4.4. Statistical Analysis

Statistical analyses were performed using Statistica (ver. 13.3; TIBCO Software Inc., Palo Alto, CA, USA). The differences in growth parameters, leaf RWC, as well as metal concentration between experimental variants were assessed using two-dimensional analysis of variance (two-way ANOVA). Analyses were followed by the multiple comparison Tukey’s HSD as a post hoc analysis. Test assumptions were checked with Levene’s test which assessed the homogeneity of variance. To explore multivariate patterns in heavy metal accumulation and growth-related traits, principal component analysis (PCA) was performed. Before analysis, all quantitative variables were standardized. PCA was conducted using the stats package in R (version 4.2.2). Separate analyses were performed for datasets containing heavy metal concentrations (Cd, Cu, Ni, Pb, and Zn) and growth traits (biomass, plant height, and cumulative shoot length). This was due to the different layout of the data, the merging of which would have resulted in the loss of parts of the data. Ellipses were determined with 95% confidence intervals.

## 5. Conclusions

This study demonstrated distinct physiological and morphological responses of two energy willow varieties (*S. × smithiana* and *S. viminalis* var. Gigantea) to heavy metal and salinity stress, applied individually and in combination. *S. × smithiana* showed better growth performance, particularly under combined metal and salt treatment. This variety exhibited more effective tolerance mechanisms, as reflected also by increased relative water content in leaves that was accompanied by metal accumulation in aboveground tissues. In contrast, *S. viminalis* var. Gigantea showed greater metal sequestration in the roots, suggesting a different stress-avoidance strategy based on the limitation of metal translocation to shoots. Salinity alone negatively affected both varieties, but its combination with heavy metals surprisingly mitigated growth-inhibitory effects, especially in *S. × smithiana*. Metal-specific uptake patterns varied by organ and treatment, with Zn and Cd showing the highest mobility and bioconcentration. This study highlights the potential of *S. × smithiana* for phytoremediation applications in mixed-contaminant environments. Further research should aim at the understanding of underlying genetic and biochemical mechanisms causing differential responses and metal tolerance in the two willow species.

## Figures and Tables

**Table 1:** Meteorological conditions at the experimental site.

Month	Parameter		
	Ambient temperature (°C)		
	min	max	mean
April	−1.8	27.2	11.1
May	2.1	27.0	17.0
June	5.9	33.7	18.8
	Air relative humidity (%)		
	min	max	mean
April	28	95	71
May	28	100	67
June	44	100	74
	Precipitation		
	time (min)	amount (mm)	days with
April	1068	27.5	14
May	1155	39.7	12
June	1112	41.2	16
	Radiation (W/m^2^)		
	min	max	mean
April	0	1264	169
May	0	1104	237
June	0	1147	224
	Light intensity (Lux)		
	min	max	mean
April	1	151,738	20,355
May	1	132,475	28,481
June	1	137,659	26,893

**Table 2:** Guideline values for heavy metal concentrations in soil used in the experiment (mg kg^−1^ dry matter) [44].

Metal	Lower Guideline Value (m1)	Higher Guideline Value (m2)
Cd	10	20
Ni	100	150
Cu	150	200
Zn	250	400
Pb	200	750

## Data Availability

Data are available from the corresponding author upon reasonable request.

## References

[1] Hou D., Jia X., Wang L., McGrath S.P., Zhu Y.G., Hu Q., Zhao F.-J., Bank M.S., O’Connor D., Nriagu J. (2025). Global Soil Pollution by Toxic Metals Threatens Agriculture and Human Health. Science.

[2] Li X., Chen Y., Kumar A., Wang Y., Singh R.P. (2024). Anthropogenic Inputs and Ecotoxicological Impacts of Heavy Metals in Aquatic Systems: A Global Review. Environ. Res..

[3] Ali H., Khan E., Ilahi I. (2019). Environmental Chemistry and Ecotoxicology of Hazardous Heavy Metals: Environmental Persistence, Toxicity, and Bioaccumulation. J. Chem..

[4] Kafle A., Timilsina A., Gautam A., Adhikari K., Bhattarai A., Aryal N. (2022). Phytoremediation: Mechanisms, Plant Selection and Enhancement by Natural and Synthetic Agents. Environ. Adv..

[5] Spinoni J., Naumann G., Carrao H., Barbosa P., Vogt J. (2014). World Drought Frequency, Duration, and Severity for 1951–2010. Int. J. Climatol..

[6] Cramer W., Guiot J., Fader M., Garrabou J., Gattuso J.P., Iglesias A., Lange M.A., Lionello P., Llasat M.C., Paz S. (2018). Climate Change and Interconnected Risks to Sustainable Development in the Mediterranean. Nat. Clim. Change.

[7] Williams A.P., Cook B.I., Smerdon J.E. (2022). Rapid Intensification of the Emerging Southwestern North American Megadrought in 2020–2021. Nat. Clim. Change.

[8] Sharma P., Jha A.B., Dubey R.S. (2024). Enhancing Phytoremediation Efficacy in Plants Cultivated in Heavy Metal-Contaminated Soil under Drought Stress: Understanding Plant Responses and Genetic Engineering Strategies. Water Air Soil Pollut..

[9] Hassani A., Azapagic A., Shokri N. (2020). Predicting Long-Term Dynamics of Soil Salinity and Sodicity on a Global Scale. Proc. Natl. Acad. Sci. USA.

[10] Stavi I., Thevs N., Priori S. (2021). Soil Salinity and Sodicity in Drylands: A Review of Causes, Effects, Monitoring, and Restoration Measures. Front. Environ. Sci..

[11] Alghamdi S.A. (2024). Drought and Salinity Effects on Plant Growth: A Comprehensive Review. SABRAO J. Breed. Genet..

[12] Karp A., Shield I. (2008). Bioenergy from plants and the sustainable yield challenge. New Phytol..

[13] Landberg T., Greger M. (2022). Phytoremediation Using Willow in Industrial Contaminated Soil. Sustainability.

[14] Pulford I.D., Watson C. (2003). Phytoremediation of Heavy Metal-Contaminated Land by Trees—A Review. Environ. Int..

[15] Cao Y., Tan Q., Zhang F., Ma C., Xiao J., Chen G. (2022). Phytoremediation Potential Evaluation of Multiple *Salix* Clones for Heavy Metals (Cd, Zn and Pb) in Flooded Soils. Sci. Total Environ..

[16] Mleczek M., Rutkowski P., Goliński P., Kaczmarek Z., Szentner K., Waliszewska B., Stolarski M., Szczukowski S. (2017). Biological Diversity of *Salix* Taxa in Cu, Pb and Zn Phytoextraction from Soil. Int. J. Phytoremediation.

[17] Nagajyoti P.C., Lee K.D., Sreekanth T.V.M. (2010). Heavy Metals, Occurrence and Toxicity for Plants: A Review. Environ. Chem. Lett..

[18] DalCorso G., Farinati S., Furini A. (2010). Regulatory Networks of Cadmium Stress in Plants. Plant Signal. Behav..

[19] Landberg T., Greger M. (1996). Differences in Uptake and Tolerance to Heavy Metals in *Salix* from Unpolluted and Polluted Areas. Appl. Geochem..

[20] Jalal A., Oliveira Junior J.C., Ribeiro J.S., Fernandes G.C., Mariano G.G., Trindade V.D.R., Reis A.R.D. (2021). Hormesis in Plants: Physiological and Biochemical Responses. Ecotoxicol. Environ. Saf..

[21] Manousaki E., Kalogerakis N. (2011). Halophytes—An Emerging Trend in Phytoremediation. Int. J. Phytoremediation.

[22] Parida A.K., Das A.B. (2005). Salt Tolerance and Salinity Effects on Plants: A Review. Ecotoxicol. Environ. Saf..

[23] Guo N., Fan L., Cao Y., Ling H., Xu G., Zhou J., Chen Q., Tao J. (2022). Comparison of Two Willow Genotypes Reveals Potential Roles of Iron-Regulated Transporter 9 and Heavy-Metal ATPase 1 in Cadmium Accumulation and Resistance in *Salix suchowensis*. Ecotoxicol. Environ. Saf..

[24] Drzewiecka K., Gąsecka M., Magdziak Z., Rybak M., Budzyńska S., Rutkowski P., Niedzielski P., Mleczek M. (2024). Drought Differently Modifies Tolerance and Metal Uptake in Zn- or Cu-Treated Male and Female *Salix × fragilis* L.. Forests.

[25] Asbjornsen H., Goldsmith G.R., Alvarado-Barrientos M.S., Rebel K.T., van Osch F.P., Rietkerk M.G., Chen J., Gotsch S., Tobón C., Geissert D.R. (2011). Ecohydrological Advances and Applications in Plant-Water Relations Research: A Review. J. Plant Ecol..

[26] Szabados L., Savouré A. (2010). Proline: A Multifunctional Amino Acid. Trends Plant Sci..

[27] Yadav S.K. (2010). Heavy Metals Toxicity in Plants: An Overview on the Role of Glutathione and Phytochelatins in Heavy Metal Stress Tolerance of Plants. S. Afr. J. Bot..

[28] Verslues P.E., Agarwal M., Katiyar-Agarwal S., Zhu J., Zhu J.K. (2006). Methods and Concepts in Quantifying Resistance to Drought, Salt and Freezing, Abiotic Stresses that Affect Plant Water Status. Plant J..

[29] Zhu J.K. (2016). Abiotic Stress Signaling and Responses in Plants. Cell.

[30] Jiang C., Wang Y., Chen Y., Wang S., Mu C., Shi X. (2024). The Phytoremediation Potential of 14 *Salix* Clones Grown in Pb/Zn and Cu Mine Tailings. Forests.

[31] Mleczek M., Rissmann I., Rutkowski P., Kaczmarek Z., Goliński P. (2009). Accumulation of Selected Heavy Metals by Different Genotypes of *Salix*. Environ. Exp. Bot..

[32] Tőzsér D., Magura T., Simon E. (2017). Heavy Metal Uptake by Plant Parts of Willow Species: A Meta-Analysis. J. Hazard. Mater..

[33] Lux A., Martinka M., Vaculík M., White P.J. (2011). Root Responses to Cadmium in the Rhizosphere: A Review. J. Exp. Bot..

[34] Liu Y., Chen G.C., Zhang J., Shi X., Wang R. (2011). Uptake of Cadmium from Hydroponic Solutions by Willows (*Salix* spp.) Seedlings. Afr. J. Biotechnol..

[35] Urošević J., Stanković D., Jokanović D., Trivan G., Rodzkin A., Jović Đ., Jovanović F. (2024). Phytoremediation Potential of Different Genotypes of *Salix alba* and *S. viminalis*. Plants.

[36] Sanità di Toppi L., Gabbrielli R. (1999). Response to Cadmium in Higher Plants. Environ. Exp. Bot..

[37] McBride M.B., Martinez C.E., Kim B. (2016). Zn, Cd, S and Trace Metal Bioaccumulation in Willow (*Salix* spp.) Cultivars Grown Hydroponically. Int. J. Phytoremediation.

[38] Ali H., Khan E., Sajad M.A. (2013). Phytoremediation of Heavy Metals—Concepts and Applications. Chemosphere.

[39] Tlustoš P., Száková J., Vysloužilová M., Pavlíková D., Weger J., Javorská H. (2007). Variation in the Uptake of Arsenic, Cadmium, Lead, and Zinc by Different Species of Willows Salix spp. Grown in Contaminated Soils. Cent. Eur. J. Biol..

[40] Kacálková L., Tlustoš P., Száková J. (2009). Phytoextraction of Cadmium, Copper, Zinc and Mercury by Selected Plants. Plant Soil Environ..

[41] Zurayk R.A., Khoury N.F., Talhouk S.N., Baalbaki R.Z. (2001). Salinity-Heavy Metal Interactions in Four Salt-Tolerant Plant Species. J. Plant Nutr..

[42] Acosta J.A., Jansen B., Kalbitz K., Faz A., Martínez-Martínez S. (2011). Salinity Increases Mobility of Heavy Metals in Soils. Chemosphere.

[43] Utmazian M.N.D.S., Wieshammer G., Vega R., Wenzel W.W. (2007). Hydroponic Screening for Metal Resistance and Accumulation of Cadmium and Zinc in Twenty Clones of Willows and Poplars. Environ. Pollut..

[44] Tóth G., Hermann T., da Silva M.R., Montanarella L. (2016). Heavy Metals in Agricultural Soils of the European Union with Implications for Food Safety. Environ. Int..

[45] Weatherley P.E., Slatyer R.O. (1957). Relationship Between Relative Turgidity and Diffusion Pressure Deficit in Leaves. Nature.

[46] Baker A.J. (1981). Accumulators and Excluders—Strategies in the Response of Plants to Heavy Metals. J. Plant Nutr..

[47] Ma L.Q., Komar K.M., Tu C., Zhang W., Cai Y., Kennelley E.D. (2001). A Fern That Hyperaccumulates Arsenic. Nature.

